# Applications of nanomaterials in tissue engineering

**DOI:** 10.1039/d1ra01849c

**Published:** 2021-05-26

**Authors:** Xinmin Zheng, Pan Zhang, Zhenxiang Fu, Siyu Meng, Liangliang Dai, Hui Yang

**Affiliations:** School of Life Sciences, Northwestern Polytechnical University Xi'an 710072 P. R. China kittyyh@nwpu.edu.cn; Institute of Medical Research, Northwestern Polytechnical University Xi'an 710072 P. R. China liangliangdai@nwpu.edu.cn

## Abstract

Recent advancement in nanotechnology has brought prominent benefits in tissue engineering, which has been used to repair or reconstruct damaged tissues or organs and design smart drug delivery systems. With numerous applications of nanomaterials in tissue engineering, it is vital to choose appropriate nanomaterials for different tissue engineering applications because of the tissue heterogeneity. Indeed, the use of nanomaterials in tissue engineering is directly determined by the choice. In this review, we mainly introduced the use of nanomaterials in tissue engineering. First, the basic characteristics, preparation and characterization methods of the types of nanomaterials are introduced briefly, followed by a detailed description of the application and research progress of nanomaterials in tissue engineering and drug delivery. Finally, the existing challenges and prospects for future applications of nanomaterials in tissue engineering are discussed.

## Abstract 2

Scientific description about the types, synthesis, functionalization, characterization application, challenges and prospects of nanomaterials in tissue engineering.

## Graphical abstract

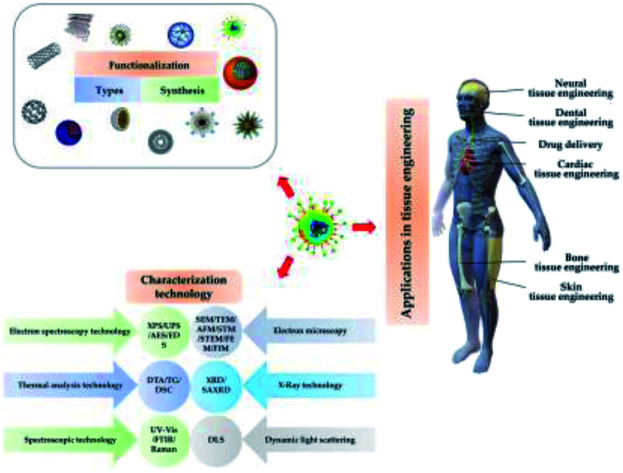

## 1. Introduction

In 2011, the European Commission (EC) defined nanomaterials as “natural, incidental or manufactured material containing particles, in an unbound state or as an aggregate or as an agglomerate and where, for 50% or more of the particles in the number size distribution, one or more external dimensions are in the size range 1–100 nm”.^1^ Different from other forms of materials, nanomaterials have unique physical (minuscule size, high surface energy, magnetic effects, large specific surface area, *etc.*), chemical (high reactivity, catalytic ability, resistance to corrosion, *etc.*) and biological properties (biocompatibility, low immunogenicity, biodegradability, *etc.*). According to the shape and morphology of nanomaterials, they can be divided into three dimensions: 0-dimensional (nanoparticles),^2^ 1-dimensional (nanowires)^3^ and 2-dimensional (nanolayers)^4^ nanomaterials, which are smaller than 100 nm in all directions, two axes, or one axis, respectively.^5^ According to their unique properties and dimensions, nanomaterials can imitate natural nanoscale extracellular matrix components and directly deliver biologically active substances. Nanoparticles (NPs) can pass through cell membranes and help cells absorb protein. Based on above considerations, nanomaterials with natural properties or functionalized with other suitable features can be constructed and applied in tissue engineering,^6^ drug delivery,^7^ bioimaging,^8^ gene therapy^9^ and other fields.

Tissue engineering is a new field of bioengineering, and it combines the techniques and principles of engineering, cell biology and material science to create tissue succedaneums that can imitate natural tissues structurally and physiologically.^10^ The National Science Foundation (NSF) officially defines “tissue engineering” as “applying the principles and methods of engineering and life sciences to fundamentally understand the structure–function relationship between normal and mammalian tissues, and develop biological substitutes to restore, maintain or improve tissue function”. That is, during tissue engineering, a variety of biomaterials (including polymers, ceramics and inorganic substances), bioactive molecules and cells are integrated to induce and/or stimulate differentiation signals, and promote tissue regeneration at the lesion or damaged site.^11^ Considering wide applications of nanomaterials in tissue engineering, the basic requirements for nanomaterials use are as follows: biodegradability, biocompatibility, biointegration, easy manufacturing and handling, and low production cost.^12^

**Fig. 1:**
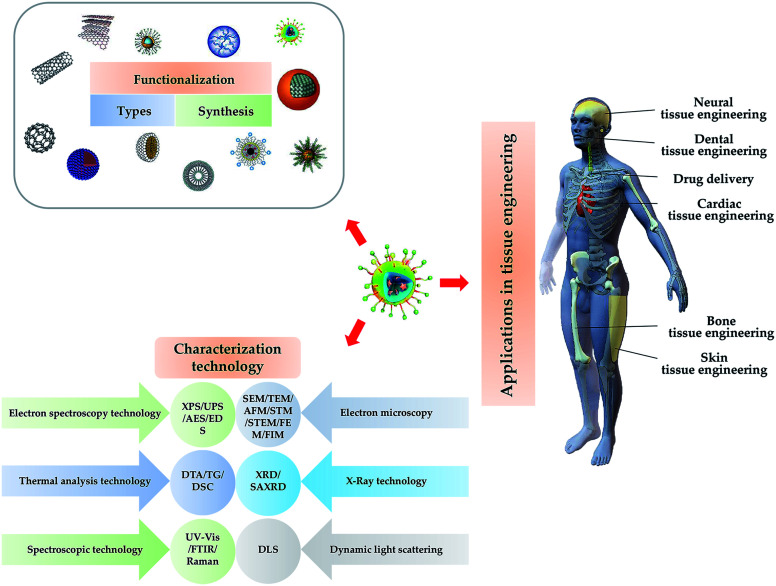
Illustration of the type, synthesis characterization and functionalization NPs and their application for tissue engineering.

This review introduces the types, synthesis, functionalization and characterization of nanomaterials used in tissue engineering, and summarizes the applications of nanomaterials in tissue engineering of bone, skin, nerve and dental, and drug delivery. A brief schematic illustration is shown in Fig. 1.

## 2. Types, synthesis, functionalization, characterization and toxicity of nanomaterials

Nanomaterials are generally prepared and synthesized using different agents, *e.g.*, metals, polymers and ceramics, *etc*. and different synthesis methods, including physical, chemical and biological, which all possess individual specialties. In order to achieve the desired functions, a series of modification approaches are always performed to especially functionalize the nanomaterials. Furthermore, with the development of science and technology, these methods are constantly updated, which reveals a more accurate understanding of nanomaterials for users. Besides, many other factors need to be paid more attention to, such as the antigenicity of the product, biocompatibility and toxicity, biodegradability, drug release behaviour, drug's physical and chemical properties, NP size, and surface charge distribution.

### 2.1. Types of nanomaterials

#### 2.1.1. Polymeric nanoparticle

**Fig. 2:**
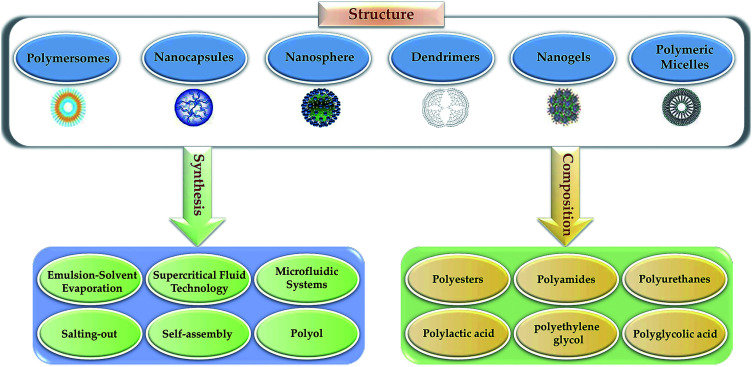
Different polymeric NPs based on composition, manufacturing process, and structure.

In recent years, polymer NPs (PNP) have attracted more and more attention. Because of the high specific surface area, the quantum size effect, modifiable characteristics, low cytotoxicity, good biocompatibility, controllable drug delivery, and maintain the bioactivity of the active agent to prevent the degradation of enzymes and other substances, PNP are gradually utilized in drug delivery.^6,13^ Generally, the individual composition and manufacturing processes generate various polymers with different structures (Fig. 2). In addition, the security and effectiveness of applications can be influenced by the relative molecular weight, polydispersity and structure.^14^ Typically, polyethylene glycol (PEG) is biocompatible, nonimmunogenic and nontoxic with a highly hydrated flexible polymer chain, and it can reduce plasma protein adsorption, renal clearance and NPs biofouling. Therefore, PEG functionalized polymers extend the half-life of drug circulation. The nanoparticle of PEGylation means PEG is added to the surface of NPs to provide a hydration layer and a space barrier around the polymer.^15^ It can reduce the nonspecific binding of serum proteins, prolong the circulation and retention time, reduce protein breakdown and renal excretion,^16^ protect the antigenic determinants from immunodetection, and thereby reducing cell clearance from the mononuclear phagocyte system. Furthermore, PEG esters have been applied to improve the pharmacokinetics of nanoformulation. Additionally, natural PNP, including albumin, polysaccharides, chitosan, and heparin, have been studied for the delivery of oligonucleotides, DNA, proteins and drugs. Meanwhile, for small molecule drugs coupled with polysaccharides such as human serum albumin^17,18^ or chitosan,^19^ their stability and biodistribution can be significantly improved. In scientific research, the commonly used PNP types are nanosphere, nanogels, polymersomes, polymeric micelles, dendrimers and nanocapsules, *etc*. The selection of different types of PNP depends on their formulation, size, shape, function, physical/chemical properties, and application purpose.

In order to obtain the suitable performance of PNP, researchers have discovered and synthesized diverse polymer materials, such as synthetic polymers polylactic acid (PLA), polyamide, PLA–glycolic acid copolymer (PLGA), polycaprolactone polyester, polyanhydride, polyglycolic acid (PGA), polysaccharides, polyurethane, polyacrylate and proteins, *etc*.,^20–22^ which all have great biocompatibility. For example, collagen is originally derived from animals with a low risk of immune rejection, can be modified by other materials for different functions.^23^ In previous studies, researchers have found that collagen has a very important role in transmitting bioactive molecules and cellular components for myocardial regeneration and repair. Therefore, collagen can act as an excellent carrier to load drugs to promote myocardial regeneration. It is also used for dermal replacement of burns and other wounds, and many *in vivo* and *in vitro* biological evaluations have been carried out.^24,25^

PLGA is another synthetic polymer with good biodegradability and biocompatibility. It can be used to load various types of drugs such as hydrophilic or hydrophobic, small or large molecule medicine. Therefore, modifying PLGA has been widely applied in the field of tissue engineering.^26^ For example, the biological activity of PLGA-based scaffolds can be improved by the bioactive glass.^27^ Typically, the material's physical compression properties and biodegradability of PGA-based fiber tubes are greatly improved by PLGA and poly-l-lactic acid (PLLA).^28^ Besides, the hydrophilicity and tensile mechanical properties of the silica-based scaffold functionalized with PLGA and gelatin gained significant enhancement as well. Moreover, *in vitro* results demonstrate that the scaffold significantly improves cell adhesion and proliferation, exhibiting a wide application potential for stem cell culture and tissue engineering.^29^

Although there are abundant polymers with individual properties, the nonimmunogenic, nontoxic, highly biocompatible, and biodegradable features should be first and commonly considered before application for tissue engineering. Notably, additional special capability, mechanical strength and hydrophilicity, are also required for tissue special engineering applications.^30^ In general, the applicability of polymeric nanoparticles must be considered comprehensively according to the type, dosage, size, durability, and exposure methods.^31^

#### 2.1.2. Metallic based nanoparticle

Metallic-based NPs are generally divided into metal NPs, metal oxide NPs and magnetic NPs. Meanwhile, metal NPs have unique antibacterial properties, as well as catalytic activity, mechanical properties and electrical conductivity, and all of these properties make them especially suitable for applications in tissue engineering.^32^ Typically, metal NPs of precious metals have been widely used in fields of cosmetics and medicine, resulting from their above-mentioned natural features. For one thing, benefiting from the good biocompatibility, facile synthesis, gold NPs have been widely used in cancer diagnosis and treatment, biological probes and drug delivery, *etc*. Zhang *et al.* synthesized gold NPs of around 20 nm diameter, which were demonstrated as efficient anti-angiogenic, inhibiting several heparin-binding growth factors, suppressing the growth of both ovarian and pancreatic tumors;^33^ similarly, silver NPs are frequently employed in biosensing, food industry, dental tissue engineering field. Xie *et al.* synthesize a novel hybrid coating adding Ag NPs, and they investigated the effect of Ag NPs on antibacterial activity. *In vivo* and *in vitro* testing indicated that the synergy between the photodynamic and physical effects of Ag NPs in the hybrid coating can kill bacterial efficiently without side-effects.^34^ Besides, other metal-based NPs have also been broadly designed for drug delivery, vaccine delivery and immune regulation, *etc*., which all benefited from its natural features described.

Metal oxide NPs have been applied in contrast agents, drug delivery, biosensors, catalyst areas as well,^35^ as there is greater freedom for the modification of the structure, size and surface chemistry properties of such NPs.^36^ For example, Hashimoto *et al.* discovered that TiO_2_ NPs could reinforce the properties of the composites, exhibiting bending strength and Young's modulus of the natural bone as well as bioactivity, thus they are being frequently employed in bone tissue engineering as advance implanting biomaterials.^37^

Magnetic nanomaterials show prominent magnetism, which is closely associated with the applied magnetic field. It has been applied in many areas, including targeting and controlling the release of drugs, increasing the growth of tissues and reducing implant infection, *etc*. Typically, magnetic nanomaterials endowed with superparamagnetic (SPM) properties including magnetite (Fe_3_O_4_), spinel ferrites (AFe_2_O_4_) and maghemite (γ-Fe_2_O_3_), not only have the characteristics of nanomaterials (such as large specific surface area, high coupling capacity and small size), but also have magnetic responsiveness and superparamagnetic properties, which gathers and locates in a magnetic field, absorb electromagnetic waves and produce heat in an alternating magnetic field. Therefore, nanomaterials can be used to deliver drugs such as doxorubicin and docetaxel to specific targets, and the temperature it produces can also be used to kill or inhibit cancer cells, and are frequently employed in much more biotherapeutic applications.^38^ For example, Sanson *et al.* synthesized modified γ-Fe_2_O_3_ magnetic NPs through a nanoprecipitation process. This formation method makes it possible for the simultaneous loading of γ-Fe_2_O_3_ and doxorubicin hydrochloride (DOX). *In vitro* testing indicated that DOX was released under local hyperthermia conditions, and γ-Fe_2_O_3_ NPs were also transmutable under a magnetic field, resulting in tumor regression.^39^

#### 2.1.3. Nanocomposite

The nanocomposite is composed of at least two or more materials, usually consisting of polymer, a filler, or an inorganic material, and it should be in the nanoscale (within 100 nm) with at least one component.^40^ Compared to the original materials, nanocomposite materials possess relatively enhanced mechanical, biodegradability and good dimensional stability through the combined synthesis advantage of organic and inorganic hybrid materials.^41^ Furthermore, nanocomposite could possess several features from their compositions. For example, graphene oxide (GO) possesses unique properties such as flexibility, biocompatibility, antibacterial activity, high surface area and ease of functionalization make it very useful in tissue engineering. Hydroxyapatite (HAP) is suitable for bone tissue engineering, and gold (Au) could induce apatite formation. Prakash *et al.* synthesized a GO/HAP/Au nanocomposite *via* the hydrothermal method, and *in vitro* results indicated that the ternary nanocomposites exhibited higher mechanical properties, good antibacterial property and excellent chemical stability and it can also improve the osteoblast cell viability, which means the potential application in bone tissue regeneration.^42^ Yang *et al.* synthesized a functionalized nanocomposite with ultra-small reduced GO (nRGO) and noncovalent PEG for the photothermal therapy of a tumor. Moreover, the power density was 0.15W cm^−2^, which was lower than that used before for the inhibition of tumors with other nanostructures, and the survival rate is remarkably improved without side effects.^43^

Additionally, there are much more types of nanomaterials, such as carbon-based nanomaterials, silicon-based nanomaterials and protein nanomaterials; considering that this review mainly focuses on nanomaterials for tissue engineering applications, these NPs are not described in this work.

### 2.2. Synthesis methods of nanomaterials

For different types of NPs, suitable synthesis methods should be selected according to their applications. There are so many affect factors as follows: first of all, the method must be simple, cheap, environmentally friendly and having a certain commercial value. Then, there should be controllability of the particle size, shape and uniformity;^44^ Finally, a series of modifications should be made to increase their stability for reducing the aggregation of particles and potential cytotoxicity of larger particles.^45^ Briefly, current synthesis methods can be divided into chemical, physical and biological methods, which are known as another classification pattern, traditional synthesis method (chemical, physical method), and green synthesis method (biological method). It is summarized in Table 1 and described in the following sections.

**Table 1:** Main synthesis methods of nanomaterials

Traditional synthesis methods		Green synthesis methods
Chemical methods	Physical methods	Biological methods
Chemical reduction	Arc discharge	Bacteria
Coprecipitation	Ball milling	Enzymes and biomolecules
Electrochemical	Evaporation–condensation	Fungi
Emulsion diffusion	Lithography	Plant extract
Polyol	Pulse wire discharge	
Salting out	Liquid–liquid interface	
Pyrolysis	Spray pyrolysis	
Thermal decomposition	Vapor and gas phase	
Sonochemical	Solvent evaporation	

#### 2.2.1. Traditional synthesis methods

##### 2.2.1.1. Thermal decomposition

The thermal decomposition method is an excellent synthesis route for the production of metallic NPs. This method involves an easy single-step process, and is cheap, not harmful to the environment, and offers higher quality metal NPs according to particle size, and size and morphology distribution.^46^ By using an appropriate surfactant to control the monodispersity of the NPs, the size and shape of the NPs can be adjusted individually.

##### 2.2.1.2. Solvent evaporation

Solvent evaporation is suitable for the formation of micelles or liposomes with the loading of insoluble drugs or those with low water solubility. Typically, polymers and hydrophobic drugs are first dissolved in organic solvents and the mixture solution is then emulsified in an aqueous solution that contains a surfactant or an emulsifier. Finally, the organic solvent can be evaporated by pressure reduction or continuous stirring and generated the resultant drug-loaded micelles or liposomes.^47^

##### 2.2.1.3. Polyol

The polyol method utilizing polyvalent alcohols with high boiling points to produce metal NPs. Polyol plays a dual role in reducing solvent and agent, which endow it the capacity to control the formation of particles. Typically, the temperature range of a high boiling point is 473–593 K, which can avoid metal solutions, forming metal NPs during the synthesis process. Finally, as a reactant, the chelating ability of polyols is conducive to preserving the key features of the reaction process.^48^

##### 2.2.1.4. Liquid–liquid interface

The liquid–liquid interface method is a direct, simple and excellent method for preparing nanocrystalline thin films through the self-assembly process. This method usually forms a nanocrystalline film at the interface of water and organic liquid. Moreover, the thickness of the film formed between the two is usually at a nanometer-scale and nonuniform, which provides a very effective method for the synthesis and self-assembly of nanocrystals.^49^

##### 2.2.1.5. Emulsion diffusion

This method meanly uses two approaches, one involves an organic solution with drug and polymer, the other, an aqueous solution with a stabilizer and solvent, which is added in the first solution mentioned above and stirred with a homogenizer. Then, adding adequate water to the solution to form NPs. Finally, the superfluous water and solvent are removed by lyophilization.^50^ The advantages of the emulsion diffusion technique include simplicity, narrow size distribution, easy scaling, homogeneity, high batch-to-batch reproducibility and high encapsulation efficiency (typically 70%).

In addition, there are many other traditional methods for nanomaterials preparation, which are not listed here. Each of them has its own advantages, and the appropriate method should be selected according to requirements.

#### 2.2.2. Green synthesis methods

**Fig. 3:**
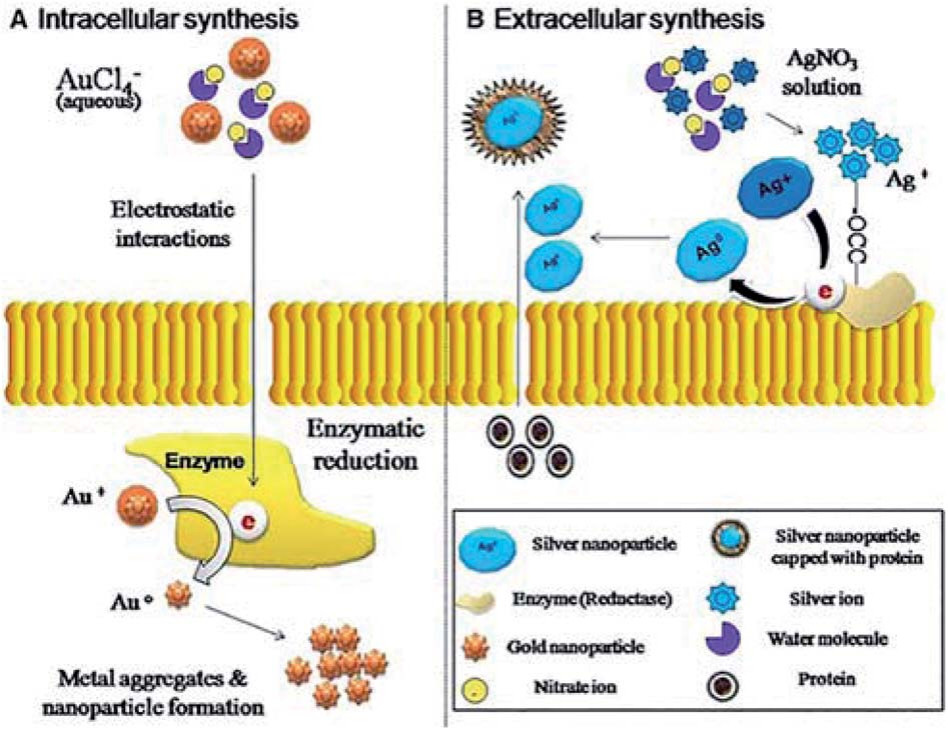
Mechanism of intracellular and extracellular synthesis of gold (Au) and silver (Ag) nanoparticles through fungi. Reprinted with permission,^53^ Copyright (2013) Springer Nature.

In recent years, a kind of green (environmentally friendly) nanotechnology has gradually caught the attention of researchers. Green nanotechnology or nanobiotechnology is the fusion of nanotechnology and biology-related technologies, which effectively avoid disadvantages of conventional physical and chemical technologies, such as toxicity, pollution, uneconomical and complicated operation, *etc*. Just like the metal NPs mentioned above, its preparation synthesized by traditional processes usually comes with many potential hazards, including environmental pollution, cytotoxicity, and carcinogenicity,^51^ which restrict their applications in tissue engineering. However, green nanobiotechnology allows metal NPs to be produced more conveniently, more cheaply and more reliably in a bio-mediated manner.^52^ Herein, we briefly introduce the biological media such as fungi, plants and bacteria for the synthesis of NPs (Fig. 3).^53^

##### 2.2.2.1. Bacteria-mediated nanoparticle generation

Some metal particles have bactericidal effects, so they are often used in food and medical fields. In previous studies, researchers discovered that sliver can accumulate in the cell wall of these bacteria up to 25% of its dry weight, and this discovery made it possible to prepare silver NPs by bacteria.^54^ For example, Saravanan *et al.* synthesized Ag NPs using *B. brevis* (NCIM 2533), and the results indicated that Ag NPs showed a drastic antibacterial properties that inhibited the multidrug-resistant pathogens. Furthermore, they also found that the protein in the extract played the role of stabilizing and capping agents, which is based on the reduction of silver ions and preventing the NPs from agglomeration.^55^

##### 2.2.2.2. Fungi-mediated nanoparticle generation

Compared to bacteria, fungi displayed the features of fast growth, easy handling & and manufacturing. Generally, the mycelial mesh produced by fungi exhibit good tolerance, resistance of airflow, pressure, fluid shearing forces *etc.*, which is helpful for the production of NPs *via* cocultivation and coprocessing approaches between precursor agents and fungi.^56^ Typically, with the abilities of metal bioaccumulation, high binding ability as well as intracellular uptake ability, and compared with bacteria, the growth rate of fungi is faster and easier to manufacture and handle in a laboratory process, fungi are increasingly produced with metallic-based NPs with good biosafety for tissue engineering. It is noted that the mechanism of forming NPs in fungi is the secretion of enzymes (such as naphthoquinones and anthraquinones), which can reduce silver ions to form NPs.^57^

##### 2.2.2.3. Plant-mediated nanoparticle generation

Compared with bacteria and fungi, the synthesis of NPs by plant extraction has its special advantages: easier to obtain, faster speed of production, safe, non-toxic and economical. Nowadays, plant extracts combined the stabilization and reduction of silver ions by biomolecules, and it provides an easier and cheaper way to produce silver NPs. In detail, the presence of photochemical substances is generally considered to be the main mechanism of plant-assisted reduction upon the process of plant production of NPs. Furthermore, plants contain a lot of steroids, saponins, carbohydrates and flavonoids, which can be used as reducing agents, with other active ingredients in plants are used as sealing agents, providing good stability for the synthesis of metal NPs.^58^

In addition, there are other green synthetic media that are not listed, such as the use of actinomycetes,^59^ algae,^60^ and yeast.^61^ Each media has its special characteristics and advantages. When producing different NPs, different preparation methods can be selected according to the needs.

### 2.3. Functionalization of nanomaterials

The interaction between NPs and cells is closely related to the regulation of the cell's behavioural function, and the distinctly first step is co-effect between the surface of NPs and cell. Therefore, proper surface functionalization and modification are vital for biomaterials, especially for the implantation of nanomaterials in the application of tissue engineering. For example, cancer cells are different from normal cells in terms of biological characteristics and microenvironment, and they have the ability to take up extracellular substances, these special characteristics act as natural targets for designing intelligent anticancer drug delivery systems.^62^ Typically, by functionalizing with tumor-targeted agents or bioimaging molecules on the surface of NPs, vehicles could effectively target tumors and deliver antitumor drugs or other functional molecules with high efficiency for tumor therapy or bioimaging.^63,64^ Our group previously constructed folic acid (FA)-functionalized dendrimer-like mesoporous drug delivery system for tumor therapy with bioimaging. This nanosystem was functionalized with FA as the targeting unit and (salicylideneimine) dicarboxylic acid (*Salphdc*) as the gatekeeper. Moreover, the introduction of *Salphdc* also endows a nanosystem with bioimaging properties. Therefore, the functionalized drug delivery system not only effectively led to tumor growth inhibition and tumor cell apoptosis with the reduced side effect, but also the *Salphdc* was used to trace the distribution *in vivo* as a fluorescent probe.^65^

### 2.4. Characterization of nanomaterials

**Fig. 4:**
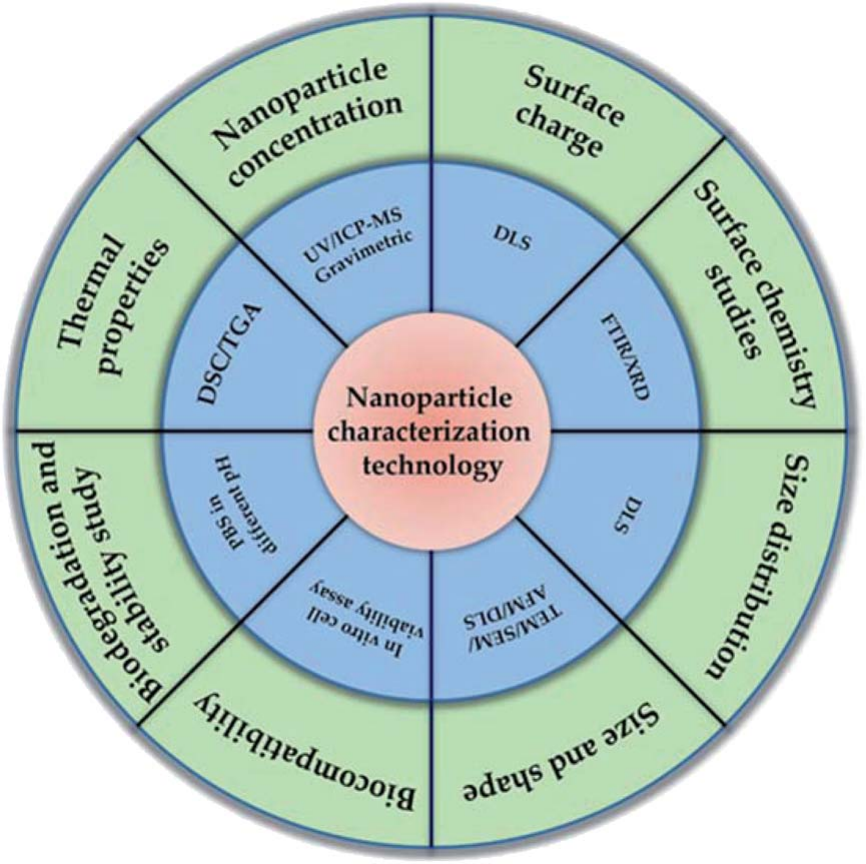
Different types of characterization techniques on nanomaterials.

Considering the functions of nanomaterials are closely related to their properties, the characterization of nanomaterials and related evaluation of properties is thus vital for nanomaterials, and should be first monitored before application. Furthermore, nanomaterial characterization is very important for understanding the controlled synthesis of NPs and their applications. Generally, accurate and reliable measurement methods (Fig. 4) are the basis of characterization. In order to better understand their properties and related functions, multiple measurement and characterization technologies must be introduced to comprehensively evaluate the properties of NPs, including charge, composition, aggregation state, size distribution, size, shape, surface chemistry and surface area of NPs. Based on above considerations, some typical characterization methods such as high-resolution scanning electron microscope (SEM), single particle inductively coupled plasma-mass spectrometry (spICP-MS), atomic force microscope (AFM), dynamic light scattering (DLS) and ultraviolet-visible (UV-vis) will be briefly discussed in this section.

### 2.5. Toxicity of nanomaterials

The toxicity of nanomaterials is closely related to their application in tissue engineering. Much more issues about biosafety are worthy of careful consideration and solving before the application, such as whether nanomaterials disturb the normal biochemical reactions in organisms? Are these changes beneficial or harmful to organs? How can this harmful effect be avoided? Generally speaking, the way in which nanomaterials react in the organism is described through the following steps: they contact the human body through subcutaneous, vein, inhalation, skin, oral administration and intraperitoneal routes; they can be absorbed after interacting with biological components (such as cells and proteins); they can spread to different organs, and maintain their structures or they can be simply changed and metabolized; they penetrate the cells of organs and they can live in cells or be released through excretion.^66^ According to these different reaction steps, we should not only consider the efficiency but also the safety before designing nanomaterials. Furthermore, from the toxicological point of view, toxic effects are affected by the following properties: size, aggregation, chemical structure, crystal properties, surface chemistry, *etc*.^67^ So it is necessary to analyse the toxicity of nanomaterials, which are used in tissue engineering and the relevant *in vivo* and *in vitro* toxicity tests should be carried out. At present, the commonly used methods to reduce or avoid the toxicity of nanomaterials are as following: selecting nanomaterials with good biocompatibility; controlling nanomaterials in the appropriate size; chemical modification (such as polyethylene glycol) on the surface of the materials; improving the structure and solubility of the materials and so on. Based on the above considerations, the purpose of reducing toxicity and enhancing therapeutic efficacy can be achieved through the above methods, so that nanomaterials can be better used in tissue engineering.

## 3. Applications of nanomaterials in tissue engineering

### 3.1. Applications of nanomaterials in dental tissue engineering

**Fig. 5:**
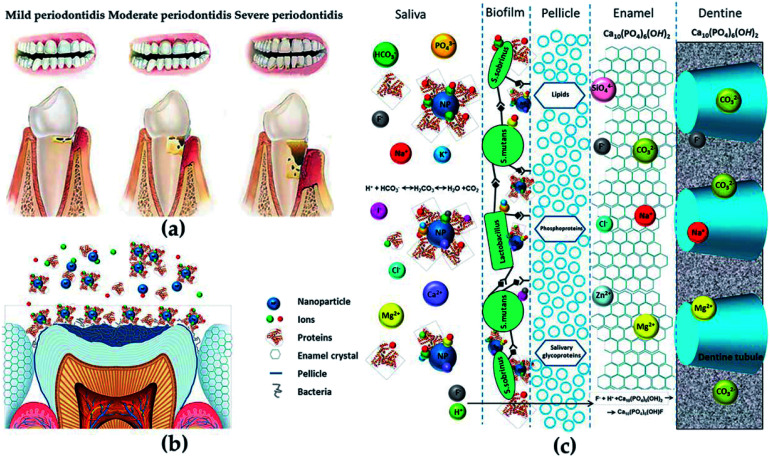
(a) Different degrees of periodontitis. (b and c) The anatomical and chemical characteristics of the potential interactions with NPs. Reprinted with permission,^71^ Copyright (2015) American Chemical Society.

Since the 21st century, the application of nanomaterials in dental tissue engineering has received more and more attention. The risk of periodontal or related diseases (such as cardiovascular disease,^68^ diabetes^69^ and rheumatoid arthritis^70^) is gradually increasing with age. Since the impairment of periodontal tissue and the loss of self-repair ability (Fig. 5a), effective treatments are needed to repair damaged tissues and restore the original structure and function in patients with periodontal diseases. Fortunately, the development of various metal and polymer nanomaterials has provided strong support for treating periodontal-related diseases (Fig. 5b and c).^71^ The applications of nanomaterials in the dental field mainly include: (1) antibacterial agents for controlling oral infections, (2) nanofillers for improving or repairing the mechanical properties and biological activities of materials used in periodontal, (3) new coatings for implants, (4) toothpaste and personal care products.

PLGA is an aliphatic polyester synthesized from PLA and PGA, with good mechanical properties, adjustable degradation rate and excellent biocompatibility. In addition, because of its biocompatibility, it has been used in research related to periodontal disease treatment.^72^ At present, the applications of PLGA-based composites in the field of dental tissue engineering are focused on the guidance of tissue regeneration, bacterial infection inhibition, periodontal drug delivery,^73^ cement formation and alveolar bone protection.^74^ Reis *et al.* constructed a PLGA-based bilayer biomaterial for the regeneration of periodontal. Compared with the control group, the bone volumetric values, trabecular thickness and trabecular number were significantly enhanced by the PLGA-based biomaterial. Moreover, new cementum and bone were only seen in the PLGA-based biomaterial group. The results indicated that the PLGA-based bilayer biomaterial possessed greater periodontal regeneration than traditional flexible membranes reported before.^75^

**Fig. 6:**
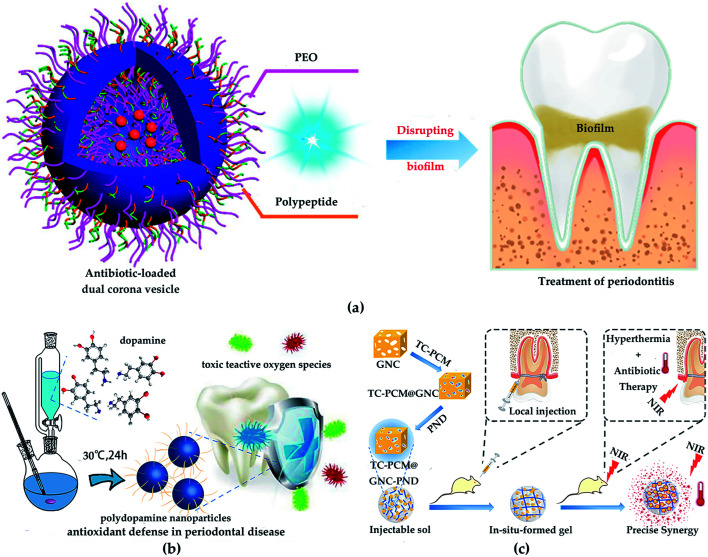
(a) Treatment of periodontitis with PCL-based vesicles. Reprinted with permission,^79^ Copyright (2019) American Chemical Society. (b) The synthesis of polydopamine NPs and their usages as efficient ROS scavengers in periodontal disease. Reprinted with permission,^80^ Copyright (2018) American Chemical Society. (c) Gold-based antibacterial strategy. Reprinted with permission,^83^ Copyright (2020) American Chemical Society.

In recent years, NPs of chitosan, silica and poly(ε-caprolactone) (PCL), *etc*. have been applied in dental tissue engineering.^76^ Boguslavsky *et al.* designed a non-destructive method for grafting monodisperse silica NPs with a diameter of 30 ± 10 nm on the surface of polystyrene, polyethylene and polyvinyl chloride. Due to the presence of silica NPs, the roughness is 1.6–2.7 times higher than that without silica NPs. Experimental results demonstrate that the bacterial attachments were significantly reduced after grafting with silica NPs, indicating that the presence of silica NPs destroyed the biofilm formation of bacteria. Regardless of the type of polymer, bacteria cannot successfully adhere to the polymer film grafted with silica NPs.^77^ Therefore, NPs are not conducive to bacterial adhesion, which effectively prevents or delays bacterial growth. Besides, chitosan possesses the properties of biodegradability and biocompatibility, which make it very popular in periodontal tissue repair. Zang *et al.* inoculated human mandibular bone marrow mesenchymal stem cells (MSCs) on chitosan-based composite scaffolds (chitosan/inorganic bovine bone) to study the treatment effect of periodontal defects. The results showed that the chitosan-based scaffold possessed great biocompatibility and improved the compressive performance of the material. Moreover, MSCs form fibrous cementum, woven/flaky bone and periodontal ligament on the chitosan-based scaffold, which exhibited a certain periodontal repair effect in the critical defect.^78^ Furthermore, benefiting from the good biodegradability, biocompatibility, and the increased permeability and retention (EPR) effect, PCL has been approved by the FDA for medical applications of dental tissue engineering. Xi *et al.* synthesized a PCL-based dual corona vesicle biofilm for the therapy of periodontitis. They found that the PCL-based biofilm possesses great antibacterial and biocompatibility properties. The *in vivo* and *in vitro* results indicated that the ciprofloxacin hydrochloride-loaded dual corona vesicle system could eradicate biofilms formed by *Escherichia coli* and *Staphylococcus aureus* strains and disrupt plaque, thereby playing a great role in the treatment of periodontitis (Fig. 6a).^79^ In addition, benefiting from the biodegradability and reductive properties, polydopamine is used to treat periodontal disease. Bao *et al.* developed a high-performance platform based on polydopamine as reactive oxygen species (ROS) killer in oxidative stress-induced periodontal disease. The *in vivo* experiment indicated polydopamine-based NPs could scavenge multiple ROS and inhibit ROS-induced inflammation reactions. Moreover, *in vitro* results showed the high efficiency of polydopamine-based NPs in removing ROS and decreasing periodontal inflammation without side effects (Fig. 6b).^80^

Because of the unique antibacterial properties and modifiability, metal nanomaterials have been recognized in dental tissue engineering. Ag, gold, TiO_2_ and ZnO are typical representatives of antibacterial metal, and their antibacterial properties can be improved through property functionalization.^31^ Besides, the size and shape of the materials may also contribute to their bactericidal activity. Studies have found that NPs with a particle size of less than 10 nm have a better bactericidal effect, and the shapes of triangular NPs have better bactericidal effects compared than spherical or needle-shaped NPs.^81^ In order to explore the antibacterial properties of Ag/Au alloy bimetallic NPs in periodontal disease, Holden *et al.* synthesized Ag/Au alloy bimetallic NPs through an electric current displacement reaction. *In vivo* experiments indicated that these NPs with good biocompatibility showed good antibacterial activity of porphyromonas gingivalis W83, which acted as a key pathogen in the development of periodontal disease, inhibited the survival of P83 plankton. Furthermore, hydrogen oxide can simulate the oxidative stress environment of chronic periodontal inflammation. When hydrogen peroxide exists, this antibacterial effect is enhanced.^82^ Zhang *et al.* synthesized a kind of light-activable nano-antibacterial scaffold based on gold nanocages to control the antibiotics release and the cooperated antibacterial effect of phototherapy and chemotherapy. Benefitting from the synergistic antibacterial effect of gold nanocages-based nano-platform, it exhibited great property of antibacterial both *in vivo* and *in vitro* (Fig. 6c).^83^

### 3.2. Applications of nanomaterials in neural tissue engineering

**Fig. 7:**
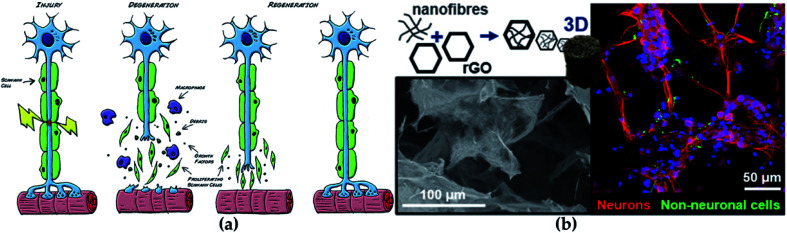
(a) The regeneration process of nerve. Reprinted with permission,^86^ Copyright (2015), with permission from Elsevier. (b) GO-based scaffolds for neural repair. Reprinted with permission,^105^ Copyright (2020) American Chemical Society.

The central nervous system (CNS) and peripheral nervous system (PNS) are the main members of the nervous system. CNS is composed of the brain and spinal cord, while PNS is composed of sensory neurons and motor neurons. Since CNS and PNS lack regenerative capacity, they often show lasting functional defects under disease or accidental injury.^84^ With the serious aging of the population, the incidence of neurodegenerative diseases is accordingly increasing, which is seriously threatening human health.^85^ At present, the existing clinical treatments against neurological diseases generally are surgical sutures, allograft and autologous transplantation, which all employed to promote the recovery of the injured nerve (Fig. 7a).^86^ Although the illness can be treated to a certain extent, there are still many disadvantages, such as immune rejection, multiple surgeries, and poor treatment effects.

The development of nerve tissue engineering brings hope to the therapy for neurological diseases. One of the typical alternative methods for repairing nerve defects is designing a reasonable nanomaterial to regulate the ECM microenvironment and cell behaviours, thus accelerating nerve regeneration. Different polymers for neural tissue engineering have been utilized, and the experimental results are exciting, containing human neural stem cell differentiation, neural gap bridging and neurite outgrowth.^87^ At present, commonly regulated agents for nerve tissue engineering include polymer scaffolds, hydrogels, NPs and nerve conduits, *etc*. No matter which material is used, its features must be met: biocompatibility, biodegradability, permeability or porosity, infection resistance, good mechanical properties and electrical conductivity.

Collagen is currently the only natural biopolymer approved for medical research on peripheral nerve regeneration.^88^ As the main component of connective tissue, collagen is distributed in various human tissues and provides structure and support for the body.^88^ There are a variety of market-oriented collagen-based nerve guide agents for peripheral nerve regeneration, such as NeuraGen®^89^ and Neuromaix®.^90^ Early experiments have found that collagen-based nerve conduits can repair small nerve gaps in primates.^91^ Furthermore, collagen combined with other substances significantly promotes the regeneration of the sciatic nerve of the rat and dog models with varying degrees.^92^ It should be noted that it is necessary to pay more attention to the source of collagen because different sources of collagen may have different effects, and the selection of collagen will determine the application.

Gelatin can be obtained by the acidic or alkaline hydrolysis of collagen. Compared to collagen, it has some unique features, including economical, high availability, hypotoxicity and biodegradability, so it has been applied in many fields such as medical video, tissue scaffold and drug delivery. Moreover, gelatin can regulate cell adhesion and proliferation and act as good implant material for tissue engineering through appropriate chemical modification.^93^ For example, the biological and dynamic properties of gelatin-based scaffolds are optimized for neural tissue engineering *via* electrospinning.^94^ Furthermore, the advantages of gelatin-based nanomaterials for neural tissue engineering could be further reinforced through functional strategies. Typically, Kriebel *et al.* prepared a composite material with a good connection between the rat sciatic nerve by functionalizing gelatin with collagen and PCL.^95^ Additionally, gelatin can also be mixed with PLA and electrospun to promote axon growth and differentiation into the motor neuronal lineage.^96^ Recently, gelatin NPs have been developed to polymer scaffolds for nerve tissue engineering with improved biocompatibility. Naseri-Nosar *et al.* found that cellulose acetate/PLA scaffolds coated with gelatin NPs had more viable cells than uncoated platforms, and they were also used as nerve guide conduits for sciatic nerve damage *in vivo* and *in vitro*.^97^

Other protein materials such as elastin, keratin, and silk are also used in nerve tissue engineering. Elastin belongs to ECM structural component, which possesses mechanical rigidity, self-assembly ability, long-term stability and biological activity; thus, elastin-based materials are majorly significant in tissue regeneration by providing elasticity to tissues and organs. However, benefiting from the biocompatibility and stability, elastin-like polypeptides are more popular in neural tissue engineering compared with elastin, widely used in therapeutic CNS diseases.^98^ Keratin polypeptide is able to produce suitable substrates and biofolding. Based on the biological properties, keratin functionalized nanomaterials enable the promotion of cell adhesion and proliferation, and their various amino acid structures can be easily modified to adapt to specific tissues as well.^99^ Electrospun polyvinyl alcohol/keratin nanofiber scaffolds allow glial cells to adhere, proliferate and survive *in vitro*, as described in other tissue engineering researches.^100^ Silk is a fibrous structural protein fabricated by silkworms and spiders, showing good elasticity, excellent biocompatibility, controllable biodegradability, antibacterial capacity and minimal immunogenicity.^101^ The multifunctional features of silk have made it suitable for biomimetic structures such as hydrogels, scaffolds, films, nanofibers and NPs. Typically, benefiting from the better structural integrity and the ability to induce axon bundles, silk-based hydrogels are commonly used in nerve tissue engineering as sustainable biomaterials. Moreover, silk hydrogels have been successfully developed to support neuronal differentiation and nerve tissue regeneration.^102^ Besides, silk fibroin also exhibits good biocompatibility and no *in vitro* cytotoxicity and can be utilized as a tissue engineering nerve conduit for potential therapy of central nervous system damage.

Carbon-based nanomaterials also play critical roles in the field of neural tissue engineering. Studies have found that carbon-based nanomaterials show great potential when interacting with neurons and neural tissue.^103^ Fullerenes, carbon nanotubes and graphene (G) are excellent representatives of carbon-based nanomaterials. Electrical stimulation is beneficial to the regeneration of neurons, which has been proven; excellent electrical conductivity, flexibility and mechanical strength cause G-based materials to perform well in neural tissue engineering, and G-based materials can also accelerate the neuron cell differentiation and proliferation.^104^ For example, the combination of functional GO nanosheets and nanofibers modulated the physicochemical and biological properties of scaffold used in repairing neural tissue and markedly improved the viability of neural progenitor cells (Fig. 7b).^105^ Benefiting from a shape similar to neurites, carbon nanotubes have become one of the most popular materials in neural tissue engineering. Moreover, a similar small size of carbon nanotube with dendrites enhances the possibility of exploring, repairing and stimulating neural networks, and it has been shown to have more potential in the treatment of neuropathy and nerve tissue damage.^103^ Additionally, good mechanical, thermal and electrical properties, make them very promising in other technical fields such as conductive composite materials and sensors.^106^

In addition, there are many materials used in the research of nerve-related diseases, such as chitosan, alginate, PLGA, PLA, PEG, *etc.* These materials have been intensively studied in nerve tissue engineering with exciting experimental results.^98^

### 3.3. Applications of nanomaterials in bone tissue engineering

Bone marrow tissue engineering is mainly developed for irreversible injuries that may require adjuvant treatment with nanomaterial-based implants. Current strategies generally include bone tissue scaffolds, physicomechanical strategy and biological strategy.^107,108^ The study of bone tissue engineering is recently focused on the exploration of 3D scaffolds, which support, strengthen, or organize the regeneration of bone tissue in a natural way.^109^ Meanwhile, benefiting from the great biocompatibility and biosafety, and the improved bone mineralization, naturally degradable nanomaterials are frequently employed for bone tissue engineering.^110^ Notably, their natural degradability makes them easily adapted for *in vivo* environment after clinical implantation and without the second operation to remove it, which reduces the patient's pain.

In bone tissue engineering, scaffold materials are commonly used to provide mechanical support for damaged parts and provide suitable conditions for bone regeneration.^111^ Ideally, a construct used for bone tissue engineering should possess appropriate mechanical properties as well as surface properties suitable for cell adhesion, proliferation and differentiation. Besides, the porosity, biological conduction, biocompatibility, and bioabsorbable characteristics of the scaffold should be considered simultaneously.^112^ Taking polymer-based scaffold as an example, which is currently recognized as one of the suitable materials for bone tissue scaffolds with poor mechanical properties. However, the mechanical properties of the stent can be well enhanced by adding nanomaterials (ceramic nanomaterials, carbon-based materials, chitosan-based nanomaterials, and metal-based NPs, *etc.*) as fillers.^113^ Furthermore, the introduction of functionalized strategies on certain nanomaterials can also regulate cell signalling pathways, change the adhesion of proteins to the scaffold as well as increase mechanical properties.^114^

As a natural biodegradable polymer with biodegradability and biocompatibility, chitosan is approved by the FDA for applications in a variety of pharmaceutical formulations.^115^ In the past few decades, chitosan has played a major role in promoting the development of bone tissue engineering.^116^ Chesnutt *et al.* developed a novel chitosan/nanocrystalline calcium phosphate scaffold based on microspheres for regenerating bone lost to disease or trauma. This composite scaffold could well support the mechanical properties and porosity for the growth of new bone tissue. Furthermore, various porous structures molded from chitosan can also stimulate bone conduction.^117^

**Fig. 8:**
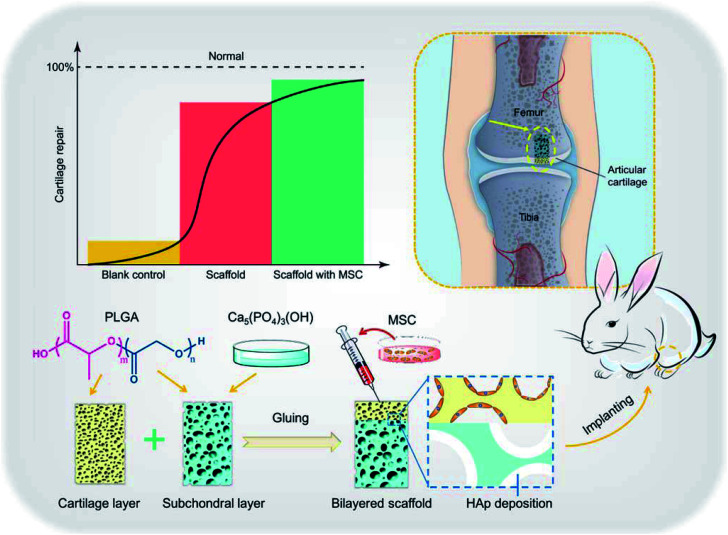
Illustration of hydroxyapatite-based scaffold-induced regeneration of bone. Reprinted with permission,^126^ Copyright (2018) American Chemical Society.

As the main inorganic component of bone tissue,^118^ hydroxyapatite NPs have received more and more attention in bone tissue engineering. Liu *et al.* constructed a chitosan/hydroxyapatite (nHAp/CTS) biomimetic nanocomposite nanofiber scaffold for evaluating the effect of bone marrow MSC mesenchymal stem cells (BMSCs) growth on nHAp/CTS for bone regeneration and exploring the molecular mechanism *in vivo* and *in vitro*. They found that nHAp/CTS scaffold could induce the proliferation of BMSCs and activate the integrin-BMP/Smad signalling pathway of BMSCs.^119^ Since the osteogenic differentiation of osteoblasts can be conducted by the chitosan/hydroxyapatite composite, its potency in bone tissue engineering has been recognized.^120,121^ Liu *et al.* studied the role of hydroxyapatite NPs in composite nanofiber/chitosan scaffolds. They found that hydroxyapatite increased cell adhesion and activated the BMP/Smad signalling pathway of bone marrow MSCs and thus promoting proliferation and bone regeneration.^119^ Compared with organic polymer NPs, they are adept at mimicking the natural inorganic phase of bone, so they are attractive biomaterial for scaffold strategy. In addition, hydroxyapatite can also be used combined with PCL,^122^ PLGA, PEG,^123^ whitlockite NPs^124,125^ and other polymer materials, which have shown good effects in bone regeneration/repair (Fig. 8).^126^

Bioactive glass–ceramic NPs (nBGC) also contribute to the development of bone tissue engineering, and they can better simulate the composition of bone than metal NPs. Singh *et al.* found that bioactive glass/polyvinyl alcohol and silk fibroin could form a double-layer scaffold through electrospinning and therefore improved the proliferation and differentiation of bone marrow MSCs.^127^ Moreover, bone marrow MSCs are conducive to the formation of new bone, while umbilical cord MSCs are conducive to the formation of new blood vessels. In addition, alginate,^128^ cross-linked dextran,^129^ PCL,^130^ PCL–chitosan (PCL–CHI),^131^ PLGA,^132^ collagen,^133^ fibrin^134^ and other materials have also been used in combination with bioactive glass to fabricate bone tissue engineering scaffolds.

Carbon-based nanomaterials can be divided into different dimensions: 0D carbon dots, fullerene and nanodiamonds, 1D carbon nanotubes, 2D graphene, 3D graphite, all of which have tunable surface functionalities, large surface area, biocompatibility, excellent mechanical strength and commercial availability.^135^ In general, the carbon-based scaffolds used for bone tissue engineering are considered as the template for the growth, proliferation, regeneration, adhesion and differentiation of bone stem cells. In terms of this, G material may be given priority, since its low metal impurity content, relatively high length and width, and simple purification process.^136^ Due to the presence of oxygen, GO has superior hydrophilicity than pure G. It is easier to disperse in water, organic solvents, and various solvents.^137^ Kumar *et al.* have constructed a kind of composite scaffold using polyethyleneimine (PEI) and GO (PEI/GO) for bone repair. Experimental results showed that PEI/GO could promote proliferation of human bone marrow MSCs and formation of their focal adhesion complexes, and induce the osteoblasts differentiation. Meanwhile, the expression of alkaline phosphatase nearly 2-fold increased, and the degree of mineralization was about 50% higher than that of GO alone. Their research shows that PEI/GO polymer composite can be used as a substitute for absorbable bioactive materials in fracture stabilization and tissue engineering of orthopedic devices.^138^ In addition, carbon nanotubes also have mechanically enhanced properties, such as high tensile strength, great electrical conductivity, and the maximum current transmittance, which have been the attraction component in enhancing nanocomposites scaffold physical properties. Of course, other types of carbon-based nanomaterials have yet been frequently used in bone tissue engineering with different degrees of biological activity.^135^

Recently, Au, Ag and titanium oxide are gradually utilized for bone tissue engineering. Because of the excellent mechanical characteristics of metal NPs, a large number of studies have used silver NPs as implants for bone tissue engineering by enhancing osteogenic properties.^139^ Pauksch *et al.* used polyoxyethylene glycerol trioleate (PGT) and polyoxyethylene sorbitol monolaurate (Tween 20) stabilized silver NPs to study the biocompatibility and osteogenic potential of Ag NPs. When silver NPs were added to the culture system of MSCs and osteoblasts, the absorption capacity of the cells was enhanced without any adverse reactions. It reveals the potential of silver NPs in bone tissue engineering.^140^ In addition, gold NPs have great potential in enhancing cell differentiation.^141^ The interaction between cells and materials could be influenced directly by the size of gold NPs. Gold NPs with a diameter of 20 nanometers have a good osteogenic effect on primary osteoblasts, whereas those with 30–50 nanometers have a significant effect on human adipose-derived stem cells.^142^ Furthermore, titanium dioxide NPs combined with a variety of polymers can also be used in the manufacture of enhanced scaffolds to study bone formation performance.^143,144^

### 3.4. Applications of nanomaterials in skin tissue engineering

Wound healing is a delicate physiological process, including hemostasis, inflammation, proliferation and remodelling/maturation.^145^ According to healing time, skin wounds generally include two categories: acute and chronic wounds.^146^ Acute wounds are featured by rupture or perforation of the skin layer, healing in a short time. Chronic wounds are usually hard to heal in the short term because they are mostly emerging as accompanied by diseases such as obesity and diabetes. Angiogenesis is an important part of the wound healing process. The vascular formation can not only provide adequate blood flow, nutrition, oxygen, *etc*.,^147^ but also accelerate the healing of wounds and base formation of granulation tissue.^148^ While abnormal blood vessel formation causes chronic wound formation, which impedes timely healing.

Therefore, when treating skin wounds, both skin tissue and vascular regeneration must be considered. For different skin injuries, there are also different treatment strategies for promoting the healing process of chronic wounds, such as local oxygen therapy, hyperbaric oxygen therapy, ozone therapy, negative-pressure wound therapy, *etc.*^149^ Typically, autologous transplantation can be used for large-area skin injuries treatment. Briefly, the full-thickness skin from other suitable parts of the donor is separated, expanded and transplanted to the wound.^150^ But this approach is limited by the donor site and the area of injury. Herein, another useful route is developed by using autologous cell-based therapy. After sufficient proliferation *in vitro*, these fused cells are used for wound healing.^151^ However, the success rate, treatment cycle and cost are extremely affected by various conditions. In order to further improve the efficiency of proliferation and biosafety, nanomaterials are used in skin tissue engineering for wound healing.^152,153^ The ideal biomaterials should meet the following criteria: (1) providing a barrier layer for regenerative keratinocytes; (2) firmly attaching to the lower dermis; (3) remodeling blood vessels at the injured site; (4) offering elastic structural support for the skin.

Chitosan is a non-toxic and biodegradable polycation polysaccharide, which can be decomposed by lysozyme *in vivo* to release harmless amino sugars.^154^ The features like good biocompatibility and cell adhesiveness make it noticeable for skin applications. The positively charged chitosan bonds to the negatively charged bacterial membrane trigger agglutination and ultimately resulted in leakage of cell components. Moreover, chitosan can chelate metals, thereby inhibiting certain enzyme activities.^155^ For example, commercially available HemCon bandage, which is invented as a hemostatic dressing, shows effectiveness from skincare application prospects.^156^ In addition, it has been confirmed that the keratin–chitosan composite film enforced the tensile properties of keratin, enhanced antibacterial property and supported the attachment of fibroblasts.^157^

**Fig. 9:**
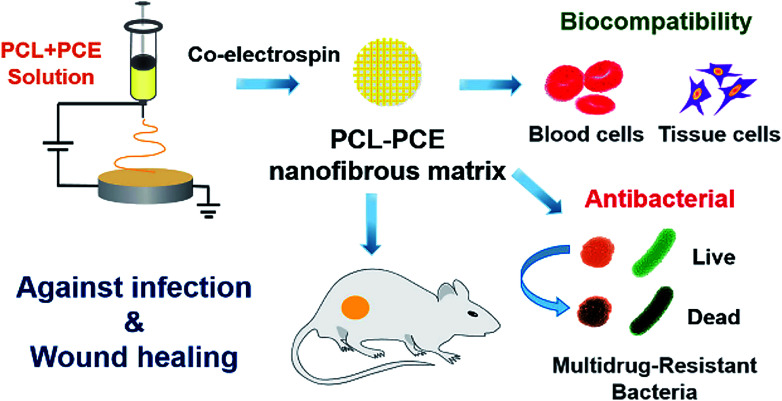
An illustration of the polypeptide-based nanocellulose composite improves skin regeneration. Reprinted with permission,^160^ Copyright (2018) American Chemical Society.

As a new type of nanomaterial, nanocellulose composed of cellulose-based nanoscale structures has attracted more and more attention. Nanocellulose has its special advantages, containing the ability to absorb wound exudate and easier remove the dressing, that traditional wound dressing materials (such as gauze) do not have, so the cellulose-based nanomaterials are also widely employed in biomedical applications for the treatment of skin diseases. Fu *et al.* discovered that nanocellulose produced by bacteria had greater advantages for wound care, such as faster healing, less inflammation, low-toxic product, and compared with traditional dressings, it helped faster tissue regeneration and enhanced the capillary formation in wounds.^158^ In addition, dressings made of nanocellulose also show great potential in the treatment of chronic ulcers of the lower extremities. The nanocellulose-based film is also used for severe burns treatment by providing a good clean environment, resulting from its excellent adhered ability of the injured area (due to its plasticity) and maintain water balance, thereby promoting wound healing. Besides, a previous study also showed that nanocellulose could reduce inflammation reactions in skin repair.^159^ Xi *et al.* developed an antibacterial, photoluminescent, and elastomeric hybrid polypeptide-based composite to suppress multidrug-resistant (MDR) bacteria and promote wound healing. The polypeptide-based nanocellulose composite showed excellent biocompatibility, biomimetic elastomeric behaviour, and robust antibacterial activity. *In vivo* results indicated that the nanocellulose composite system could efficiently inhibit MDR bacteria-derived wound infection and prominently improve skin regeneration (Fig. 9).^160^ Additionally, nanocomposite material-introduced nanocellulose, such as polyethylene glycol,^161^ polyvinyl alcohol,^162^ chitosan,^163^ gelatin,^164^ alginate,^165^ could also exhibit an advanced skin tissue repair effect.

In order to promote revascularization in skin tissue engineering, designing suitable nanocarriers for gene transfection can effectively protect molecules from nucleases and regulate gene release during gene delivery therapy. Mayo *et al.* synthesized PLGA NPs loaded with anti-vascular endothelial growth factor (VEGF) interceptor plasmid pFlt23K for the treatment of neovascular disorders. They successfully modulated gene expression (and decreased the secretion of vascular endothelial growth factor from epithelial cells), showing the potential to treat traumatic diseases.^166^ In terms of skin regeneration, stem cells with high expression of vascular endothelial growth factor and transient modification have been developed to promote angiogenesis (especially after transplantation).^167^ According to reports, biodegradable nanomaterials can deliver the epidermal growth factor gene to human MSCs and cells derived from human embryonic stem cells. Meanwhile, the treated cells are more vigorous to produce epidermal growth factor, and better implant effects are observed in target tissues. Compared with the control cells, angiogenesis was enhanced 2–4 times when the nanostructured scaffolds were implanted with VEGF expressing stem cells. It exhibits the clinical potency of stem cells engineered with biodegradable NPs for skin tissue regeneration.^168^

### 3.5. Applications of nanomaterials in drug delivery

Tissue engineering is to create, repair, and/or replace tissues and organs by using cells, biomaterials and bioactive molecules applied individually or in combination. However, the survival rate of cells is poor during the first few days after transplant,^169^ furthermore, the combination of cells and growth factors or drugs to promote the survival rate of cells has also been proved a failure.^170^ Drug delivery system could enhance the efficiency and safety in tissue engineering; thus, it is particularly important to establish an effective drug delivery system to guide the functional tissue regeneration *in situ* without harmful effects on the rest of the body.^171^ But it remains remarkably challenging, including the limited understanding of the biological barriers and it induces poor drug delivery efficiency and bioavailability *in vivo*. Biomaterials improve the delivery efficacy of a series of drug compounds *via* loading or co-conjugation approaches, including enzymes, vaccines, drugs, peptides, and antibodies. Although it has made significant progress, there are still challenges for enhanced delivery efficiency and disease therapy effects. In the emerging field of nanomaterial-based drug delivery, the range of available materials has been expanded, including dendrimers, polymeric nanospheres, liposomes, lipid NPs, micelles and inorganic nanomaterials (such as iron oxide, gold, metal, and silicon). Meanwhile, biomaterials have been designed to initiate drug release *in situ* in response to a series of environmental stimuli (for example enzymes, pH, glucose, pressure, temperature).^172^ Herein, we take polymer micelles and dendrimers as examples to introduce the application and progress of nanocarriers in drug delivery and disease therapy of tissue engineering.

**Fig. 10:**
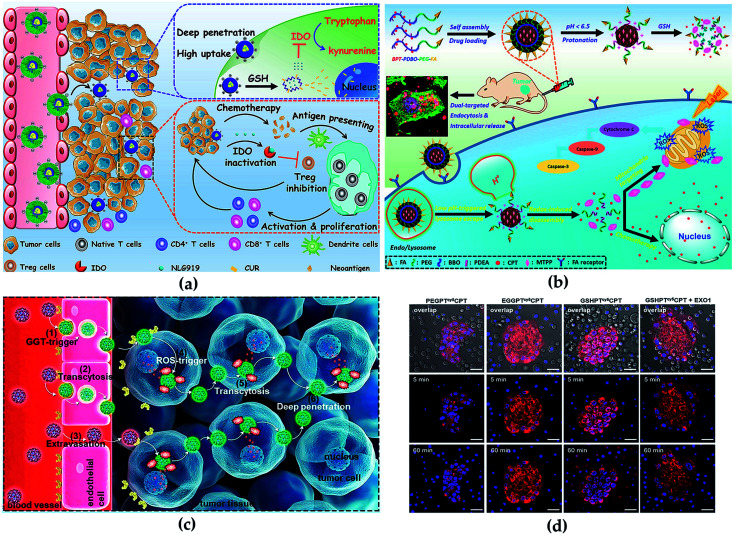
(a) Synthesis routes of micelle and illustration of antitumor *in vivo*. Reprinted with permission,^174^ Copyright (2020), with permission from Elsevier. (b) Illustration of cascade-responsive disassemble micelles with dual-targeting capability for tumor therapy *in vivo*. Reprinted with permission,^175^ Copyright (2017) American Chemical Society. (c) Illustration of the CPT conjugate for PDA therapy. Reprinted with permission,^178^ Copyright (2020) American Chemical Society. (d) Transcytosis of the conjugates using the co-incubation method visualized. Reprinted with permission,^178^ Copyright (2020) American Chemical Society.

Polymer micelles are generally formed by self-assembly and emulsion evaporation methods. Because of its unique physical and chemical properties, micelle nanosystems are conducive to effective drug delivery for disease therapy. Notably, the critical micelle concentration value also plays a vital role in the process of polymer micelle administration. Lower critical micelle concentration value endows micelles with higher pharmacokinetic stability.^173^ In the previous study, our group constructed a modifiable drug delivery micelle with improved tumor endocytosis and penetration for antitumor and loaded immune checkpoint IDO inhibitor NLG919, which enabled the redox/pH cascade-responsive release. The *in vivo* and *in vitro* results indicated that the micelle overcame biological barriers, enhanced antitumor immune response and inhibited tumor growth, metastasis and recurrence (Fig. 10a).^174^ Furthermore, we synthesized a micelle-based drug delivery system with dual-targeting potential (targeting mitochondria and cell), which enhanced the distribution of drugs on cell and subcellular levels to improve antitumor efficacy. The micelle was endocytosed by tumor cells improved by the FA receptor-mediated pathway. The *in vivo* and *in vitro* results indicated that the polymeric micelle drug delivery system could effectively improve both the targeted delivery efficiency and the combinational antitumor efficacy with very low side effects (Fig. 10b).^175^

Typical advantages of dendrimers are highly branched multivalent properties, enhanced solubility and reduced drug toxicity.^176^ Unique dendrimer structures provide various opportunities for interaction with guest molecules, which include the covalent coupling of the drug to the terminal group of the dendrimer, or the physical binding of the inner cavity induced by hydrogen bonding, hydrophobic attachment and electrostatic interaction, thus significantly improving drug loading content, which benefits disease therapy with high efficiency. In detail, a direct relationship between algebra and packaging trends has been proposed previously: the more dendrimers are generated, the more the number of functional groups, and therefore, the larger the space for drug loading.^177^ Besides, the covalent binding of guest molecules with the surface group of dendrimers can promote the formation process of dendrimer–drug conjugates. Wang *et al.* constructed a dendrimer–camptothecin (CPT) conjugate by combined camptothecin and polyamidoamine (PAMAM) dendrimers with a ROS-sensitive linker and modification of the surface with glutathione for the treatment of pancreatic ductal adenocarcinoma (PDA) (Fig. 10c). *In vivo* and *in vitro* results indicated that the CPT conjugate had the transcytosis of drug conjugates from one cell to another (Fig. 10d) and improved the penetration properties within the tumor parenchyma and exhibited high antitumor activity.^178^ Linking the modified cisplatin with dendrimers by chemical bonds, Li *et al.* synthesized stimuli-responsive clustered NPs for antitumor. The NPs were more concentrated in tumor sites and releasing drugs, and effectively inhibited the growth of the tumor.^176^

In addition, many other nanomaterials have also been proven to have great properties and efficiency for drug delivery and disease therapy, such as metal-based nanomaterials, carbon-based nanomaterials and organic polymers. All of these materials exhibit the potential in drug loading and delivery for the therapy of tumor or other diseases.^179^

## 4. Challenges and future perspectives

In this review, we discussed the basic characteristics, preparation and characterization methods of different types of nanomaterials, and their typical applications in tissue engineering. The development of nanomaterials and their applications in tissue engineering are very important for the repair or regeneration of destructed tissue. With regard to existing nanotechnology, more and more researchers try to develop new biomaterials using different combinations of numerous nanomaterials. Moreover, when these nanomaterials are used in tissue engineering to replace damaged organs, issues of the sensitivity of implanted materials, the subsequent immune response, the potential toxicity, the impact on reproduction and even the impact on fetal development, *etc.*, have to be carefully considered. The development of new nanomaterials provides an excellent opportunity for tissue engineering, which must satisfy the expectations of patients and the needs of clinicians. Although nanomaterials may bring undoubted benefits to medicine, the application of these man-made nanomaterials also have nonnegligible health risks. The risk must be minimized according to the precautionary principle during the development, testing and clinical applications of these materials. This still needs to take a big step in the biosafety, utilization and stability of nanomaterials. In the future, we believe the development of nanotechnologies that are rationally designed will solve most of the problems encountered in current tissue engineering.

## Author contributions

Data curation, X. Z., P. Z., Z. F., S. M.; writing-original draft preparation, X. Z.; writing-review and editing, L. D.; supervision, H. Y.; project administration, L. D.; funding acquisition, L. D. and H. Y. All authors have read and agreed to the published version of the manuscript.

## Conflicts of interest

There are no conflicts to declare.

## Supplementary Material
